# Molecular evolution of Drosophila Sex-lethal and related sex determining genes

**DOI:** 10.1186/1471-2148-12-5

**Published:** 2012-01-14

**Authors:** Charles Mullon, Andrew Pomiankowski, Max Reuter

**Affiliations:** 1 Department of Genetics, Environment and Evolution, University College London, Darwin Building, Gower Street, London WC1E 6BT, UK; 2 Centre of Mathematics and Physics in the Life Sciences and Experimental Biology, University College London, Physics Building, Gower Street, London WC1E 6BT, UK

## Abstract

**Background:**

Sex determining mechanisms are evolutionarily labile and related species often use different primary signals and gene regulatory networks. This is well illustrated by the sex determining cascade of *Drosophila *fruitflies, which have recruited *Sex-lethal *as the master switch and cellular memory of sexual identity, a role performed in other insects by the gene *transformer*. Here we investigate the evolutionary change in the coding sequences of sex determining genes associated with the recruitment of *Sex-lethal*. We analyze sequences of *Sex-lethal *itself, its *Drosophila *paralogue *sister-or-Sex-lethal *and downstream targets *transformer *and *doublesex*.

**Results:**

We find that the recruitment of *sister-or-Sex-lethal *was associated with a number of adaptive amino acid substitutions, followed by a tightening of purifying selection within the *Drosophila *clade. Sequences of the paralogue *sister-or-Sex-lethal*, in contrast, show a signature of rampant positive selection and relaxation of purifying selection. The recruitment of *Sex-lethal *as top regulator and memory gene is associated with a significant release from purifying selection in *transformer *throughout the *Drosophila *clade. In addition, *doublesex *shows a signature of positive selection and relaxation of purifying selection in the *Drosophila *clade. A similar pattern is seen in sequences from the sister Tephritidae clade.

**Conclusions:**

The pattern of molecular evolution we observe for *Sex-lethal *and its paralogue *sister-or-Sex-lethal *is not characteristic of a duplication followed by neo-functionalization. Rather, evidence suggests a sub-functionalization scenario achieved through the evolution of sophisticated splicing. As expected, we find that *transformer *evolves under relaxed purifying selection after the recruitment of *Sex-lethal *in *Drosophila*. Finally, the observation of *doublesex *adaptation in both *Drosophila *and Tephritidae suggests that these changes are due to ongoing adaptation of downstream sex-specific regulation, rather than being associated the recruitment of *Sex-lethal *and the resulting change in the topology of the sex determining cascade.

## Background

Sex determination is the process by which an individual makes the developmental decision to become male or female. Unlike other fundamental processes in development, such as body patterning by *Hox *genes [1], the molecular mechanisms responsible for sex determination have not been conserved [2]. Instead, a plethora of sex determining strategies exist, varying greatly in the primary signal used in sex determination. This diversity can be seen across the Diptera alone, where the initial signal is genetic in *Drosophila melanogaster*, environmental in *Sciara ocellaris *and maternal in *Chrysomya rufifacies *[3,4, for reviews]. Variation and fast turnover also occur in the genetic implementation of sex determining mechanisms. The housefly *Musca domestica *provides a striking example for evolutionary lability at this level. In some populations, male development is triggered by the presence of masculinizing alleles with varying genomic location in some populations, whereas in other populations these factors are fixed and sex is based on the presence of a dominant feminizing allele at another locus [5].

**Figure 1:**
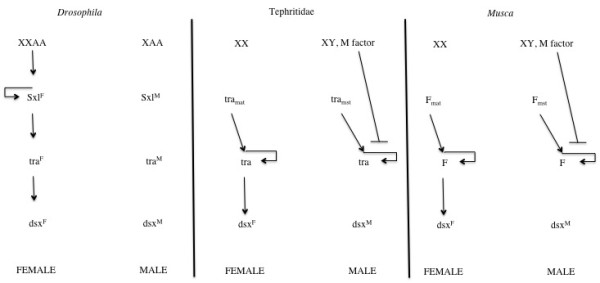
**Sex determination networks in flies**. A comparison between the sex determination networks in the *Drosophila*, Tephritidae and *Musca domestica *(after [3]).

Dipteran sex determination probably provides the best studied model for understanding the evolution of sex determining mechanisms. Particularly well described is the genetic cascade of *D. melanogaster*, in which sex is determined by a primary signal that is transmitted through a short cascade of regulatory genes and translated into sexual phenotypes via downstream transcription factors (see Figure 1) [6, for a most recent review]. In *D. melanogaster*, the primary signal is provided by a gene counting mechanism sensing the number of X chromosomes (2 in females, 1 in males). This primary input is translated into differential expression of splice forms of the switch gene *Sex-lethal *(*Sxl*). Female embryos express a fully functional SXL protein while males produce a shorter peptide that lacks an RNA-binding domain. The female protein SXLF maintains the master signal through an auto-regulatory self-splicing loop. At the same time, SXLF transmits the female signal further down the cascade by ensuring that *transformer *(*tra*) transcripts are spliced into a female-specific, functional, form. The female TRAF protein, in turn, forms a heterodimer with TRA2 protein to regulate the splicing of the transcription factor *doublesex *(*dsx*) mRNA. The resulting female variant DSXF regulates female differentiation of somatic tissue. In males, the truncated SXLM has no regulatory effect, leading to the production of an equally inactive default splice variant of *tra*. The presence of TRAM (i.e., absence of TRAF), results in the production of default male forms of the downstream target *dsx*, DSXM. *tra *also regulates the splicing of another transcription factor fruitless. A sex-specific mRNA of this gene is produced in males that contributes to differentiation of male nervous tissue.

A comparison between the *Drosophila *sex determining cascade and those of the closely related families Tephritidae and *Muscidae *(Figure 1) illustrates how sex determining cascades evolved from the bottom up [7]. The downstream genes *tra *and *dsx *are used by all three groups. Only *Drosophila *uses the switch gene *Sxl *which appears to have been recruited recently to the top of the cascade. The ancestral condition is present in the Tephritidae and *Muscidae*, which uses *tra *and a *tra*-orthologue, respectively, as the switch gene [8-10]. The *tra *gene in these species maintains its signal through a self-splicing loop operated by the TRA/TRA2 heterodimer. This mechanism is common among the Diptera [8] and might be an ancestral element of the sex determining cascade across the insects [11], as indicated by the discovery in honeybees of a conserved gene with homology to *tra *[12]. Outside the insects, there is no evidence for *tra *involvement in sex determination. Homologues of the downstream target *dsx*, however, have been identified not only in other insects [5,13] but also in worms and mammals [14,15]. This suggests that *dsx *has been involved in sex determination for a very long time [16].

It is unclear what general principles underlie the bottom-up evolution of sex determining mechanisms or whether indeed such general principles exist [17,18]. However, adaptive scenarios have been proposed that provide plausible adaptive scenarios for the the recruitment of *Sxl *to the *Drosophila *cascade [16]. In this paper, we investigate the molecular changes to the *Drosophila *sex determining cascade due to the recruitment of *Sxl*. We use sequences from twelve *Drosophila *species, a sample of species from the Tephritidae, as well as *Musca domestica *to infer patterns of selection on the coding regions of sex determining genes. Thanks to the detailed molecular knowledge of sex determination in *D. melanogaster *and the simple structure of the genetic cascade, we are able to formulate clear hypotheses for the consequences of recruitment of *Sxl *on the molecular evolution of *Sxl *itself and its downstream targets.

**Figure 2:**
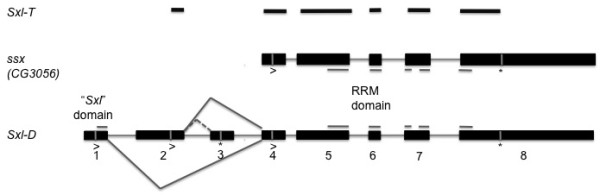
**Structure of Drosophila and tephritid Sex-lethal (Sxl-D and Sxl-T in the Figure) and the Drosophila paralogue ssx**. The Figure shows splice variants of *Sxl-D*, the position of translation start sites (>) and stop codons (*) as well as the position of the *Sxl*-specific and RRM protein domains following [47]. The gene structure for *Sxl*-T is for indicative purposes only, as only exonic sequences are available and the exact position of introns is unknown.

Hypotheses about the patterns of molecular evolution in *Drosophila Sxl *can be derived from the evolutionary origin of the gene. Evidence suggests that the recruitment of *Sxl *coincided with a gene duplication event [19,20] that gave rise to *Sxl *and its paralogue *CG3056*, now named *sister-of-Sex-lethal *(*ssx*) [20]. Both *Drosophila *genes and their orthologue in the Tephritidae contain two RNA recognition motifs (RRM domains) [[19], see also Figure 2]. *Drosophila Sxl *encodes an additional N-terminal protein domain, the '*Sxl*-specific domain' (Figure 2). Truncated proteins lacking this domain show the same binding affinity as the full *Sxl *protein, but fail to induce female-specific self-splicing of *Sxl *transcripts [21]. The presence of the *Sxl*-specific domain in *Drosophila*, together with the fact that neither *ssx *in *Drosophila *nor the *Sxl *orthologue in the Tephritidae and *Muscidae *show sex-specific expression or splicing [19,22-25] suggest neo-functionalization of the *Drosophila Sxl *duplicate [19]. According to this hypothesis, the common ancestor of Drosophilidae and Tephritidae would have employed a sex determining mechanism similar to that used by the Tephritidae today [16]; following duplication in the *Drosophila *lineage, *Sxl *then adapted to its new role in sex determination while the paralogue *ssx *retained the ancestral, non-sex specific function. Based on this scenario, we would expect a signature of adaptation under positive selection in *Drosophila Sxl *but comparable levels of purifying selection on tephritid *Sxl *and *Drosophila ssx*.

A recent study has put forward an alternative scenario for the evolution of *Sxl *and *ssx *[20], whereby *Sxl *would have acquired a new role in sex determination while retaining its ancestral, sex-independent function, whereas *ssx *would have neo-functionalized to take on roles not previously performed by *Sxl*. This scenario is based on the observations that loss of *ssx *had no significant negative effect in fly viability or fertility combined with the discovery of a conserved, non-sex-specific splice variant of *Sxl*. Under this scenario, we would expect signals of positive selection in both *ssx *and *Drosophila-Sxl*, while tephritid *Sxl *would have evolved under purifying selection.

We also predict an effect of *Sxl *recruitment on the evolution of the downstream genes in the sex determining cascade. In *Drosophila, Sxl *took over the memory function previously held by *tra*. This should have led to evolutionary change at two levels. First, we expect relaxation of selection on amino acids involved in the now obsolete self-splicing of *tra*. Whether this will result in changes in the *tra *coding sequence depends on the degree to which the self-splicing mechanism differs from the interaction of TRA/TRA2 with its regulatory targets *dsx *and *fru*. The high degree of similarity between TRA/TRA2 binding sites in the intronic sequences of *tra *outside of *Drosophila *(the target of self-splicing) [26-28] and in *dsx *[29] and *fru *[30] within and outside of *Drosophila *(the targets of allo-splicing) suggest similar splicing mechanism. The evolutionary loss of *tra *self-splicing in *Drosophila *then might not have resulted in changes in its amino acid sequence. However, there is also evidence that the self-splicing mechanism involves a protein complex including not only TRA/TRA2 and RBP1 but also an as yet unknown factor [28, named X-SR]. TRA coding regions involved in the interactions with these proteins would then be free to erode after *Sxl *recruitment rendered *tra *self-splicing redundant. Second, we expect adaptive change to accommodate the new splicing regulation of *tra *through *Sxl*. As this regulation in *Drosophila *occurs via the binding of SXL to a non-coding region of *tra *transcripts, adaptation of *tra *is expected to have occurred at the level of non-coding (intronic) rather than coding sequences. Adaptive evolution in response to the recruitment of *Drosophila Sxl *is not expected at the bottom gene of the cascade, as *dsx *does not directly interact with *Sxl *and the functional link between *tra *and *dsx *is unaffected by *Sxl *recruitment. If at all, the recruitment of *Sxl *might have allowed fine-tuning of the sex-specific signal of *dsx *in *Drosophila *[16], which would be evident in its relative expression in males and females rather than in changes in the coding sequence.

## Results

### Molecular evolution of Sxl

We analyzed patterns of molecular evolution by applying phylogenetic maximum likelihood models implemented in PAML [31] to sequence alignments of sex determining genes (see Methods section for details). The mode of selection acting on coding sequences (purifying, neutral or positive) was inferred by estimating the *ω *= *dN/dS *ratio that compares the rates of non-synonymous and synonymous mutations. An *ω *ratio smaller than one indicates that sequences are under purifying selection, where non-synonymous mutations are eliminated from the gene-pool and hence fixed at a lower rate than synonymous mutations; an *ω *ratio equal to one occurs in neutrally evolving sequences where drift affects synonymous and non-synonymous mutations to the same extent; finally, an *ω *ratio greater than one occurs in sequences under positive selection, where non-synonymous mutations have a greater chance of reaching fixation than synonymous mutations.

**Figure 3:**
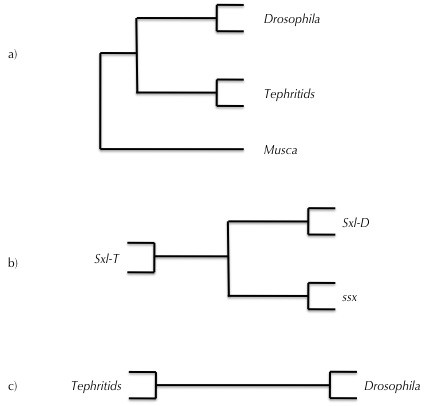
**Illustration of the phylogenetic trees used for analyses of molecular evolution**. a) Analyses including sequences from *Drosophila *Analyses including sequences from *Drosophila*, the Tephritidae and *M. domestica*, b) the Tephritidae and a *Drosophila *paralogue, as used for *Sxl *and *ssx*, and c) analyses including sequences from *Drosophila *and the Tephritidae.

We first inferred selection on *Sxl *associated with its recruitment to the sex determining pathway of *Drosophila *by analyzing an alignment of *Sxl *sequences from the *Drosophila *species, the Tephritidae and *M. domestica *(Figure 3a, Additional File 1, Figure S1). Before analyzing evolutionary patterns specifically associated with *Sxl *recruitment, we tested for global patterns of neutral evolution and positive selection along all branches of the tree (Test 1, see Methods). We detected a proportion of amino acids that evolve neutrally (Table 1, line a), but there was no evidence for the evolution of amino acids under positive selection across all taxa studied (P = 1, Additional File 2, Table S1).

**Table 1:** Significant likelihood ratio tests of selection on Sxl in Drosophila, the Tephritidae and M. domestica sequences

Test	Line	**Alternative M**^***a***^	**Null M**^***a***^	2Δ*L*	df	**P**^***b***^	**Sites**^***c***^
1	a	Nearly Neutral	One ratio	112.53	1	< 0.0001	21
2-D	b	Local selection	Local relaxation	9.16	1	0.0024	17
2-T	c	Local relaxation	Uniform Selection	262.18	2	< 0.0001	1
*2-T*	*d*	*Local selection*	*Local relaxation*	*5.46*	*1*	*0.019*	*0*
*3-D*	e	*Local relaxation*	*Uniform Selection*	*248.25*	*2*	*< 0.0001*	*0*
3-R^*d*^	f	Local relaxation	Uniform Selection	208.30	2	< 0.0001	43

^*a *^Alternative and null models, see Additional File 2, Table S1 for more information on models and Log-likelihood values, ^*b *^P value calculated from a *χ*^*2 *^distribution, ^*c *^number of sites significant in Bayesian post-hoc tests (P < 0.05), ^*d *^clade consisting of all species excluding *Drosophila*. The alignment, after deleting gaps, was composed of 298 codons. Tests that we deemed weakly significant because Bayesian post-hoc tests did not detect relevant AA are shown in italics.

We then looked for signatures of selection during *Sxl*'s recruitment to the sex determining cascade. We tested for a signal of relaxed selection on the basal branch leading to the *Drosophila *clade, i.e., identifying amino acids that evolve neutrally on the basal branch but are under purifying selection on the rest of the tree. This test was significant (*P *< 0.0001, Additional File 2, Table S1) revealing an evolutionary shift from purifying selection to neutral evolution on the branch leading to the *Drosophila *clade. Given the signature of relaxed purifying selection, we then tested for the signal of positive selection on the basal *Drosophila *branch, seeking to identify sites that are under positive selection on that branch but evolve neutrally or are under purifying selection on the rest of the tree. We found significant evidence of positive selection (*P *= 0.0024, Table 1, line b). Furthermore, posterior Bayesian analysis provided evidence for adaptive fixation of 17 amino acids (with *P *≥ 95%) (Table 1, line b). Taken together, these tests indicate that the recruitment of *Sxl *to the *Drosophila *sex determining cascade coincided with release from selective constraint and adaptive changes in the protein sequence.

As a comparison, the same tests were applied to assess selection specific to the basal branch of the tephritid clade. The test for positive selection was significant (Table 1, line d), but Bayesian analysis did not identify any site under positive selection (Table 1, line d). The failure to identify selected codons by Bayesian estimation does not provide reliable evidence for positive selection on the branch leading to the Tephritidae. Inconsistent results of this type can occur whenever codons cannot be unambiguously allocated to a particular class of sites (Z. Yang, pers. comm.). Our data therefore provide, at best, weak evidence for positive selection at the root of the Tephritidae, in contrast to strong evidence for positive selection at the root of the *Drosophila *clade.

The previous tests investigated the selective signatures of substitutions along the branch coinciding with *Sxl*'s recruitment to the sex determining cascade. We also performed tests to investigate patterns of evolutionary change following the recruitment to sex determination. A first test sought to identify sites that are under relaxed selection along all branches of the *Drosophila *clade but under purifying selection elsewhere in the tree. This test was significant (*P *< 0.0001, Table 1, line e), but again no individual amino acid was identified by site-specific Bayesian tests. Evidence for relaxed selection of *Sxl *in the *Drosophila *clade is therefore inconclusive. In contrast to this, we obtained highly significant results for the mirror model, which identified amino acids that are under purifying selection in *Drosophila *but evolve neutrally across the rest of the clade. Moreover, Bayesian posterior tests provided robust evidence for relaxation of purifying selection affecting 43 sites (Table 1, line f). Tests for positive selection either along the internal branches of the *Drosophila *clade or the rest of the tree were non-significant. Together this evidence suggests that the main evolutionary change to *Sxl *after its recruitment to *Drosophila *sex determination was a relative strengthening of purifying selection. The absence of recurrent positive adaption within the *Drosophila *clade indicates that adaptive change of *Sxl *to its new role in sex determination occurred prior to the divergence of the *Drosophila *species.

### Molecular evolution of the Sxl paralogue ssx

We investigated selection pressures associated with the duplication of *Sxl *in *Drosophila *by analysing an alignment including *Drosophila Sxl *and *ssx *as well as their orthologue *Sxl *in the Tephritidae (Figure 3b, Additional File 3, Figure S2). Analysis of selection on specific sites along all branches provided evidence for neutrally evolving sites over the whole tree (Table 2, line a) but the test for tree-wide positive selection was not significant (*P *= 1, Additional File 4, Table S2). Branch-site models on the branch leading from the *Sxl/ssx *split to the *ssx *clade in *Drosophila *provided evidence for the adaptive fixation of 18 amino acids on the ancestral branch (Table 2, line b). In addition, the test for local relaxation across the *ssx *clade, rather than the basal branch only, was significant (Table 2, line c) and identified 31 codons that evolve under purifying selection in *Sxl*, but neutrally in *ssx*. So we find evidence from two different tests: adaptive fixation of some amino acids on the ancestral branch of *ssx *(from the first test) which is followed by neutral evolution of some amino acids in the clade (from the second test). Because nine of the 18 amino acids that were inferred by Bayesian analysis to have been positively fixed at the *Sxl/ssx *split were also found to evolve neutrally once fixed in the *ssx *clade, they are likely characteristic of *Sxl *evolution rather than *ssx *evolution. There remains consistent evidence of nine amino acids fixing under positive selection for *ssx*. Our results suggest that adaptive evolution following the gene duplication in *Drosophila *was not restricted to *Sxl*, as extensive ancestral adaptive evolution was observed for amino acids of the paralogue *ssx*.

**Table 2:** Significant likelihood ratio tests for selection on Drosophila and tephritid Sxl and Drosophila ssx

Test	Line	**Alternative M**^***a***^	**Null M**^***a***^	2Δ*L*	df	**P**^***b***^	**Sites**^***c***^
1	a	Nearly Neutral	One ratio	189.21	1	< 0.0001	24
2-*ssx*	b	Local selection	Local relaxation	7.94	1	0.019	18
3-*ssx*	c	Local relaxation	Uniform Selection	193.70	2	< 0.0001	35

^*a *^Alternative and null models, see Additional File 4, Table S2 for more information on models and Log-likelihood values, ^*b *^P value calculated from a *χ*^*2 *^distribution, ^*c *^number of sites significant in Bayesian post-hoc tests (P < 0.05). The alignment, after deleting gaps, was composed of 265 codons.

### Molecular evolution of downstream sex determining genes

We performed analyses designed to detect changes in the pattern of molecular evolution of the downstream sex determining genes *tra *and *dsx*, coinciding with the recruitment of *Sxl *in *Drosophila*. For *tra*, we analyzed an alignment of *Drosophila *and tephritid sequences (Figure 3c, Additional File 5, Figure S3). We found evidence for site-specific neutral evolution (Table 3, line a). The likelihood ratio test for local relaxation on the basal branch (separating the *Drosophila *clade and the Tephritidae) was significant, but no amino acid was found to have evolved neutrally on that branch (Table 3, line b), so the overall evidence for relaxation on the basal branch alone is weak. Tests of local relaxation of selective constraint were significant for both clades (Table 3, lines c and d). The effect was quantitatively stronger in the *Drosophila *clade than in the Tephritidae (Additional File 6, Table S3); 16 sites were inferred to evolve neutrally in *Drosophila*, but only 1 in the Tephritidae. Taken together, these results show that the recruitment of *Sxl *to the sex determining cascade coincided with a significant loosening of selective constraint in the *Drosophila *clade.

**Table 3:** Significant likelihood ratio tests of selection on transformer in Drosophila and the Tephritidae

Test	Line	**Alternative M**^***a***^	**Null M**^***a***^	2Δ*L*	df	**P**^***b***^	**Sites**^***c***^
1	a	Nearly Neutral	One ratio	13.75	1	0.0002	4
*2*	*b*	*Local relaxation*	*Uniform Selection*	*5.39*	*2*	*0.02*	*0*
3-D	c	Local relaxation	Uniform Selection	64.89	2	< 0.0001	16
3-T	d	Local relaxation	Uniform Selection	15.79	2	< 0.0001	1

^*a *^Alternative and null models, see Additional File 6, Table S3 for more information on models and Log-likelihood values, ^*b *^P value calculated from a *χ*^*2 *^distribution, ^*c *^number of sites significant in Bayesian post-hoc tests (P < 0.05). The alignment, after deleting gaps, was composed of 122 codons. Tests that we deemed weakly significant because Bayesian post-hoc tests did not detect relevant AA are shown in italics.

The evidence for a relaxed purifying selection in *Drosophila tra *is corroborated by the pattern of insertions and deletions (indels) for *tra *that is not taken into account by PAML's analysis of coding sequences. First, the coding sequence of the *tra *protein is on average much shorter in *Drosophila *than in the tephritids (Table 4). Whilst some indels appear to be species-specific, we observe four substantial domains (length greater than 30 nucleotides, with a total of 469 nucleotides) that are conserved in all tephritid species but absent in all *Drosophila *species (see Additional File 6, Figure S3). These represent indel events that have most likely taken place on the ancestral branch dividing the two clades. The difference in mean coding length between the two clades is 652 nucleotides, so the 469 ancestral indels make up a significant share of this length difference. These important structural changes in the protein provide further evidence for the relaxation of purifying selection on *tra *coinciding with the recruitment of *Sxl *in the sex determination network.

**Table 4:** Coding sequence (CDS) length and indel rate within the Drosophila and tephritid clades for transformer

Clade	CDS Length		**Indel rate**^***a***^	
	Mean	Variance	Mean	Variance
*Drosophila*	603	4412	0.409	0.397
Tephritids	1255	132	0.258	0.062
P Value	< 0.0001	< 0.0001	0.017	< 0.0001

^*a *^Indel rate was calculated for each pair of species within a clade by dividing the number of indel sites by the number of nucleotides in the pairwise alignment, then further dividing by the branch length between the two species estimated using the *dsx *gene.

In addition to a general shortening, we observe much greater variance in the length of the *tra *protein between *Drosophila *than between tephritid species (see Table 4). This again suggests weaker purifying selection against indels, or less consistent selection across *Drosophila *species. The comparison between *Drosophila *and the Tephritidae is potentially confounded by differences in branch length (i.e., divergence time) between the clades. To control for this effect, pairwise comparisons were made within each clade, and the number of indels per site was scaled by the branch lengths separating each pair of species. Based on these data, we found that the rate of indels is higher in the *Drosophila *than the tephritid clade (Wilcoxon test, *W *= 1092, *P *= 0.017). In addition, the variance in the indel rate was much higher in the *Drosophila *than the tephritid clade (Bartlett test for homogeneity of variances, *K*^2 ^= 28.6, *P *< 0.0001). From a statistical point of view these tests are not entirely rigorous, as they do not take into account the inter-dependence between the data points derived from overlapping pairs of species. However, the large difference observed, in particular in the variance in indel rates, suggests that the evolutionary processes are not identical in the two clades, with lower evolutionary constraint in the *Drosophila *clade.

We finally analyzed patterns of molecular evolution in the *dsx *gene. The lower rate of change in *dsx *allowed us to include the gene sequence from *M. domestica *in our analysis, without removing an excess of amino acids due to alignment gaps (Figure 3a, Additional File 7, Figure S4). As with *Sxl *and *tra*, analyses based on site models revealed that some sites evolve neutrally across the entire tree (Table 5, line a), but there was no evidence for consistent positive selection (*P *= 1, Additional File 8, Table S4). Including the sequences from *M. domestica *allowed us to root the split between the *Drosophila *and tephritid clades.

**Table 5:** Significant likelihood ratio tests of selection on doublesex in Drosophila, the Tephritidae and M. domestica

Test	Line	**Alternative M**^***a***^	**Null M**^***a***^	2Δ*L*	df	**P**^***b***^	**Sites**^***c***^
1	a	Nearly Neutral	One ratio	183.62	1	0.0001	17
2-D	b	Local selection	Local relaxation	10.52	1	0.005	6
2-T	c	Local selection	Local relaxation	8.34	1	0.015	4
3-D	d	Local relaxation	Uniform Selection	36.64	2	< 0.0001	4
3-R^*d*^	e	Local relaxation	Uniform Selection	70.17	2	< 0.0001	8

^*a *^Alternative and null models, see Additional File 8, Table S4 for more information on models and Log-likelihood values, ^*b *^P value calculated from a *χ*^*2 *^distribution, ^*c *^number of sites significant in Bayesian post-hoc tests (P < 0.05). The alignment, after deleting gaps, was composed of 364 codons.

Applying tests to infer changes in selection on the basal branches leading to the *Drosophila *and tephritid clades, we detected evidence for positive selection along both branches (Table 5, lines b and c), with 6 and 4 sites being identified as targets in *Drosophila *and the Tephritidae, respectively. Comparing the evolution of the gene within and outside of *Drosophila*, we found evidence for relaxation of purifying selection at a small proportion of sites within *Drosophila *(4 sites, Table 5, line d) and in the outgroup (8 sites in the Tephritidae and *M. domestica*, Table 5, line e).

### Type I error in the inference of positive selection

Although our analyses provide evidence for adaptation at some point in the phylogeny of every gene except *tra*, caution is required when inferring past selection from DNA sequences. When sequences are very divergent, the occurrence of multiple substitutions at a site (saturation) can cause the rate of synonymous substitutions (*dS*) to be under-estimated. This, in turn, results in an inflated *dN*/*dS *ratio and the inference of spurious positive selection. Problems of this kind are unlikely to affect our results because the MLE methods used here estimate the most likely *dN/dS *ratio based on patterns of substitutions along all branches of a tree and have been shown to be significantly more powerful and reliable for inferring ancestral positive selection than counting methods comparing pairs of sequences [32-34].

In order to formally rule out effects of saturation on our results, we performed extensive simulations in an approach previously taken by Studer et al. [34, see also Methods]. These simulations seek to estimate the type I error in a conservative scenario. We generated artificial alignments by simulating sequence evolution along the tree of the original sequences using the parameters of the null models (in the absence of positive selection) for all genes. To make the test conservative, the risk of saturation was artificially increased by multiplying the number of substitutions per codon on the tested branch by a factor of 1.5. For each gene, a set of 200 simulated alignments was analyzed for positive selection using the same tests as in the original analyses. The highest rate of false positives observed in our conservative approach was 1% (for *Sxl*), indicating that our inferences of positive selection are extremely unlikely to be due to type I error.

## Discussion

In this paper we investigated the changes in the patterns of molecular evolution evolution of sex determining genes associated with the recruitment of *Sxl *to the top of the *Drosophila *sex determining cascade. We analyzed the evolution of *Sxl *itself, its *Drosophila *paralogue *ssx*, and the downstream targets *tra *and *dsx*, using sequences from species of *Drosophila *and their sister clade the Tephritidae, as well as *M. domestica*.

*Drosophila Sxl *is thought to have originated through duplication on the branch leading to the *Drosophila *clade [19,20]. The ancestral function of *Sxl*, and its current function in the Diptera outside *Drosophila *are not known to be associated with sex determination [22,24]. Two hypotheses have been put forward as to how new and ancestral functions were shared between the two *Drosophila *paralogues *Sxl *and *ssx*. Traut *et al*. [19] proposed that *Sxl *neo-functionalized to its sex determining role whereas the paralogue *ssx *would have maintained the ancestral functions. Alternatively, Cline *et al*. [20] suggested *Sxl *would take on a new sex determining function while simultaneously both *Sxl *and *ssx *would sub-functionalize to share non sex-specific functions ancestrally performed by *Sxl*.

Based on our analyses and including previous findings, it is now possible to weigh up the relative merits of these two evolutionary scenarios. The fact that *Sxl *has undergone significant changes is not contentious. It is clear that the gene has adapted to its new sex determining role by the addition of a new domain and the evolution of sophisticated RNA splicing. Our analyses have shown that *Sxl *has undergone adaptive evolution in its coding sequence at a limited number of amino acids, followed by a tightening of purifying selection on the protein sequence. It seems furthermore likely that *Sxl *has retained an ancestral function, an interpretation that is supported by the fact that one of the *Sxl *transcripts in *Drosophila *lacks the *Sxl*-specific domain and is expressed in both sexes [20]. But in the light of our findings it is now also clear that *ssx *has undergone adaptive evolution. Thus, we have shown that the gene shows a signature of adaptive change as well as a release from purifying selection on its coding sequence, resulting in a protein that differs significantly from both its paralogue in *Drosophila *and its orthologue in the Tephritidae. This finding is in line with Cline *et al*.'s [20] hypothesis of sub-functionalization. Adaptation in both genes could further indicate that the duplication of *Sxl *allowed for the alleviation of 'adaptive conflict' [35] previously imposed by the double function of the ancestral gene. Establishing whether this is the case, however, will require more detailed information on the non sex-specific functions of *Drosophila Sxl *and *ssx *and their orthologue in other dipteran species.

Our analyses were also able to shed some light on the repercussions of *Sxl *recruitment in the patterns of molecular evolution of genes further down the sex determining cascade. The protein evolution observed in *Drosophila tra *is characterized by extensive neutral evolution and high rates of indels. These results echo those found by a previous study using a smaller number of species [36]. The evidence for sequence degradation adds to the inferred loss of the putative auto-regulation domain in *Drosophila tra *[11,28], and corroborates the view that the recruitment of *Sxl *as the main sex switch gene relieved the pressure of purifying selection on *tra*. Whether the relaxation of selection on *Drosophila tra *outside the specific auto-regulatory domain is due to the loss of the sexual memory function is difficult to ascertain. The TRA/TRA2 binding sites in *Drosophila dsx *and *fru *are well conserved [26-30], implying that TRA's regulatory function is still required. There are, however, suggestions that the auto-regulation of *tra *is more complicated than its regulation of *dsx *[28,37]; rather than forming an enhancing complex with TRA2 as for *dsx *pre-mRNA, the TRA protein silences expression in *tra *pre-mRNA. Regions of the protein only involved in these specific auto-regulatory mechanisms would be free to erode after recruitment of *Sxl *in *Drosophila*.

There is also the additional (and non-exclusive) possibility that the relaxation of purifying selection on *tra *sequence is the result of *Sxl *taking over other sex-specific regulatory functions. Over thirty potential functional binding sites for *Sxl *have been found in *Drosophila *[38,39], some of these may have been ancestrally regulated by *tra*. The loss of these functional links from *tra *could have relieved it from selection pressure. Since *Drosophila Sxl *was sex specifically spliced by *tra *before it was promoted to top regulator in the sex determining cascade [40], there has been a relatively long evolutionary time for *Sxl *and *tra *to exchange various functions, potentially selected for their effectiveness of specific target splicing. In that light it would be interesting to compare the putative targets of *Sxl *in *Drosophila *with those of *tra *outside of *Drosophila*. Overlap between these two sets would support this hypothesis.

Taken together, our results indicate that the adaption of *tra *to its new regulatory role in somatic sex determination (loss of self-regulation, and potential targets, interaction with *Sxl*), did not require positively selected amino acid substitutions, but rather the degradation of redundant parts of the protein-coding sequence. This partial erosion was complemented with selective changes elsewhere in the gene sequence. Thus, we observe changes in the non-coding sequence, where we see the emergence and conservation of a *Sxl *binding site in intronic sequences of *Drosophila tra *(data not shown).

The evolution of *Sxl *and *tra *in *Drosophila *can be compared with a different change in the top regulator in honeybees. In this group, female development is driven *complementary sex determiner *(*csd*), a switch gene specific to the genus *Apis*. Sex determination in honeybees is haplodiploid, with females heterozygous and males hemizygous at the *csd *locus. Similar to *Drosophila Sxl, csd *arose by duplication of *feminizer *(*fem*), the ancestral top regulator and orthologue of *tra *[12,41]. In contrast to *Drosophila*, where *Sxl *underwent a short bout of adaptation on its recruitment and *tra *shows evidence of relaxed selection, *csd *in honeybees has undergone continued positive selection since its creation by duplication, whereas *fem *has experienced tightening purifying selection. Presumably, it is the requirement for heterozygosity in females that drives continued change in the amino acid sequence of *csd *[41]. The strong purifying selection on *fem *has been attributed to potentially deleterious effects of unspecific protein-protein interactions that could arise from amino acid changes [41]. Our results suggest that such deleterious effects either play a lesser role in *Drosophila *or are compensated by the benefit of mutations degrading *tra *functions that have become redundant since the recruitment of *Sxl*.

We also found evidence for positive selection and relaxed purifying selection in *dsx*, the transcription factor translating the sex determining signal into sex-specific gene expression and differentiation. This was detected both in the *Drosophila *and in the Tephritidae (albeit in different amino acids). Furthermore, a preliminary analysis found evidence for positive selection in *fruitless *(*fru*), a gene with a similar position to *dsx *in the sex determining cascade that is directly regulated by *tra *(data not shown). The evidence for widespread adaptive evolution in the downstream target genes of sex determination in *Drosophila *is surprising as neither *dsx *nor *fru *interact with *Sxl *and both should therefore be unaffected by the recruitment of *Sxl*. In the Tephritidae, adaptive change is even more surprising, as it occurs in the absence of any (known) topological change in the sex determining cascade. The results therefore suggest that although *dsx *is conserved in function and sequence across a large part of the animal tree [42], continuous evolutionary change occurs independent of topological changes in the network. It is unclear what forces might generate positive selection on downstream sex determining genes [16].

## Conclusions

In this study, we have shown that the recruitment of *Sxl *to the *Drosophila *sex determining cascade has coincided with changes in the evolution of the *Sxl *gene itself, its paralogue *ssx *and the downstream genes involved in sex determination, *tra, dsx *and *fru*. Studying a well-known and relatively simple gene cascade has enabled us to relate and confront the evolution of a network structure with the direction of selection on the amino acids of the genes participating in that network. Patterns of molecular evolution of amino acids in relation to network changes (or indeed their absence) in *Drosophila *emerge from our analysis, notably the sub-functionalization of *Sxl *and *ssx*, and the degeneration of *tra*, along with the ongoing evolution of *dsx *in *Drosophila *and the Tephritidae. Future experimental work will hopefully shed more light on this issue, notably by investigating the molecular function of *Sxl *splice forms that are produced equally in both sexes and so may perform one the of the ancestral function of the gene.

## Methods

### Sequence data

For the genus *Drosophila*, our analyses were based on the genome sequence and annotation of *D. melanogaster *[43] and genome assemblies for eleven additional species, *D. simulans, D. sechelia, D. yakuba, D. erecta, D. ananassae, D. pseudoobscura, D. persimilis, D. willistoni, D. virilis *and *D. grimshawi*. Starting from the *D. melanogaster *annotation, we identified orthologous sequences of *Sxl, ssx, tra, fru *and *dsx *in the eleven other species by querying their genomic scaffolds with exonic sequences of *D. melanogaster *using the BLAST program (v8.11.0) [44].

Orthologues of the genes in the Tephritidae were obtained from the NCBI sequence repository. In these searches, we used the female splice variants of *Sxl *and *tra *in *D. melanogaster *and concatenated the early and late variants of *Sxl*. For *dsx*, the male and female variants were also concatenated. Using this approach, we obtained orthologues of *Sxl *from one *Ceratitis *and one *Bactrocera *species, and orthologues of *tra *and *dsx *from eight *Anastrepha*, one *Ceratitis *and three *Bactrocera *species. The accession numbers of these sequences can be found in Additional File 9, Table S5. For the gene *fruitless*, alignments of available sequences produced only a moderate number of overlapping sites. This gene was therefore excluded form our analyses.

Sequences were aligned with the Mafft software (v6.624 beta) [45] using the E-INS-i option with default parameters. Exon boundaries were checked for the *Drosophila *species using the Jalview visualization software (v11) [46] and the DEDB database [47]. Before proceeding with selection analyses, all positions containing indels were removed from the alignment. Complete alignments are provided in the Additional Files accompanying this article (Additional File 1, Figure S1, Additional File 3, Figure S2, Additional File 5, Figure S3 and Additional File 7, Figure S4).

### Maximum likelihood tests of positive selection

Estimations of the selection pressure on coding sequences were based on the ω *= dN/dS *ratio, comparing the rates of non-synonymous and synonymous mutations. We estimated *ω *ratios using PAML software (v4.4b) [31]. Several different types of maximum likelihood tests of positive selection were performed.

Test 1 aims to detect amino acids that are under positive selection on all branches. It assumes that codons are under identical selection pressures on all branches of the tree (*ω*^*T *^*= ω*^*B *^= *ω*^*D *^for each codon, see Figure 3 for a tree with branch labels). Test 1 is based on the three "sites" models [31]: the "one ratio" model [31] estimates a single *ω*_0 _value for all codons, the "nearly neutral" model ("M1a") classifies codons into those under purifying selection (for which it estimates an *ω*_0 _< 1) and those evolving neutrally (for which it fixes *ω*_1 _*= *1), and finally the "positive selection" model ("M2a") adds a third category of codons under positive selection (for which an *ω*_2 _> 1 is estimated). Likelihood ratio tests were used to detect relaxation of purifying selection (comparing the likelihood of the nearly neutral model to that of the one-ratio model) and positive selection (comparing the positive selection to the nearly neutral model). These tests compare the difference in likelihood between two nested models (as 2Δ*L*) to a *χ*^*2 *^distribution with degrees of freedom equal to the difference in the number of parameters used by the two models compared.

Tests 2 and 3 are based on "branch-site" models [48] and are aimed at detecting differences in the selective pressures that affect particular codons on particular branches of the tree. Test 2 allows us to detect selective pressures on the basal branch between the *Drosophila *and tephritid clades, coinciding with the recruitment of *Sxl *to the *Drosophila *sex determining cascade. It identifies amino acids that either evolve neutrally on the basal branch but are under purifying selection in both the *Drosophila *and tephritid clades (*ω*^*T *^= *ω*^*D *^< 1, *ω*^*B *^= 1) or those that evolve under positive selection on the basal branch while being under purifying or no selection within the clades (*ω*^*T *^= *ω*^*D *^≤ 1, *ω*^*B *^> 1). Test 3 detects general changes in the mode of selection following the recruitment of *Sxl*. It allows us to detect amino acids that are under purifying selection in one clade but evolve neutrally in the rest of the tree, or those that evolve neutrally in one clade but are under positive selection on the rest of the tree. Each of these tests are specified by three models. The null model ("uniform selection") does not include differences between branches and considers two classes of sites, those evolving under purifying selection (*ω*_0 _< 1) and those evolving neutrally (*ω*_1 _= 1) across the whole tree. This model is identical to the "nearly neutral model" of test 1 ("M1a"). The first alternative model ("local relaxation") assumes relaxed selection on the branch(es) to be tested. It includes a third class of sites that are evolving neutrally (with *ω*_1 _*= *1) on the tested branch(es) while being under purifying selection (with *ω*_0 _< 1) on the remainder of the tree. The second alternative model ("local selection") omits the class of branch-specific neutral evolution of the "local relaxation" model and replaces it by two additional classes in which sites are under positive selection (with *ω*_2 _> 1) on the tested branch(es) but are either under purifying selection (with *ω*_0 _< 1) or evolve neutrally (with *ω*_1 _*= *1) on the rest of tree. Again, likelihood ratio tests are used to assess the improvement of fit between increasingly more parameter-rich models. Whenever likelihood ratio tests provided evidence for significant positive selection, a bayesian procedure [48] implemented in PAML was used to identify the individual sites that most likely were the targets of that selection. All tests were performed according to PAML guidance [31].

To check that saturation of synonymous substitutions was not spuriously inflating the *dN/dS *ratio, we performed a simulation analysis following the approach of [34]. Artificial alignments were produced with EVOLVER [31] under the null model of "local relaxation". All parameters were set at values equal to the maximum likelihood estimates obtained by fitting the "local relaxation" model to the original data, except the length of the tested branch (defined as number of substitutions per codon in EVOLVER) which was multiplied by a factor of 1.5. The resulting alignments were tested for positive selection by applying test 2. The log-likelihood difference (2Δ*L*) of these tests was recorded. As the sequences were generated in the absence of true positive selection but with longer branch lengths, this procedure provided a null distribution of 2Δ*L *for sequences with exaggerated divergence against which we tested the value observed in the analysis of the original data. Due to the artificially increased branch lengths in the simulated data, this approach provides an extremely conservative test for positive selection. If the test on the original sequences was prone to type I error due to saturation in the estimated rate of synonymous substitutions, then tests on the even more divergent produced alignments should be even more so, and the original 2Δ*L *value would be unlikely to fall within the extremes of the null distribution.

## Authors' contributions

MR and AP conceived the study, CM performed the analyses, all authors wrote the manuscript. All authors read and approved the final manuscript.

## Supplementary Material

Additional file 1 **Figure S1. Alignment of Sex-lethal of *Drosophila *species, the Tephritidae and *M. domestica***. Alignment used for analyses of *Sex-lethal *including sequences from *Drosophila *species, the Tephritidae and *Musca domestica*. The alignment is shown translated into amino acids. Sites under relaxed selection in the Tephritidae and *Musca *are indicated by a "1" in the line "Clade R *ω *= 1", those under positive selection on the basal brach leading to *Drosophila Sex-lethal *are indicated by a "*" in the line "Clade Droso *ω *> 1". These site-specific results are based on Bayes Empirical Bayes analyses mentioned in the main text. The RRM domains of the protein are also shown. Click here for file

Additional file 2 **Table S1. Maximum likelihood models of selection on *Sxl *in *Drosophila*, the Tephritidae and *M. domestica *sequences**. Click here for file

Additional file 3 **Figure S2. Alignment of Sex-lethal of *Drosophila *species and the Tephritidae only, and *ssx *of *Drosophila***. Alignment used for analyses of *Sex-lethal *and *ssx *including sequences from *Drosophila *species and the Tephritidae. The alignment is shown translated into amino acids. Sites under relaxed selection in *ssx *(or *CG3056*) are indicated by a "1" in the line "Clade CG *ω *= 1", those under positive selection on the basal brach leading to *ssx *are indicated by a "*" in the line "Clade CG ω > 1". These site-specific results are based on Bayes Empirical Bayes analyses mentioned in the main text. The RRM domains of the protein are also shown. Click here for file

Additional file 4 **Table S2. Maximum likelihood ratio models for selection on *Drosophila *and tephritid *Sxl *and *Drosophila ssx***. Click here for file

Additional file 5 **Figure S3. Alignment of transformer of *Drosophila *species and the Tephritidae only**. Alignment used for analyses of *transformer *including sequences from *Drosophila *species and the Tephritidae. The alignment is shown translated into amino acids. Sites under relaxed selection in the tephritid clade are indicated by a "1" in the line "Clade Teph *ω *= 1", those under relaxed selection in the *Drosophila *clade are indicated by a "1" in the line "Clade Droso *ω *= 1". These site-specific results are based on Bayes Empirical Bayes analyses mentioned in the main text. The domains that are conserved in the Tephritids but absent in *Drosophila *and longer that 10 AAs are from position 2 to 99, 215 to 249, 295 to 317 and 370 to 380 of A. *serpentina*. Click here for file

Additional file 6 **Table S3. Maximum likelihood models of selection on *transformer *in *Drosophila *and the Tephritidae**. Click here for file

Additional file 7 **Figure S4. Alignment of doublesex of *Drosophila *species, the Tephritidae and *M. domestica***. Alignment used for analyses of *doublesex *including sequences from *Drosophila *species, the Tephritidae and *Musca domestica*. The alignment is shown translated into amino acids. Sites under positive selection on the basal brach leading to the tephritid clade are indicated by a "*" in the line "Clade Teph *ω *> 1", those under positive selection on the basal brach leading to the *Drosophila *clade are indicated by a "*" in the line "Clade Droso *ω *> 1". These site-specific results are based on Bayes Empirical Bayes analyses mentioned in the main text. Male- and female-specific domain of the protein are also shown. Click here for file

Additional file 8 **Table S4. Maximum likelihood models of selection on *doublesex *in *Drosophila*, the Tephritidae and *M. domestica***. Click here for file

Additional file 9 **Table S5. GI Accession numbers for sequences**. Click here for file
