# Supplementary figures and images for: Molecular evolution of Drosophila Sex-lethal and related sex determining genes
Source: BMC Evol Biol. 2012 Jan 14;12:5. doi: 10.1186/1471-2148-12-5 (PMC3292462; doi:10.1186/1471-2148-12-5)

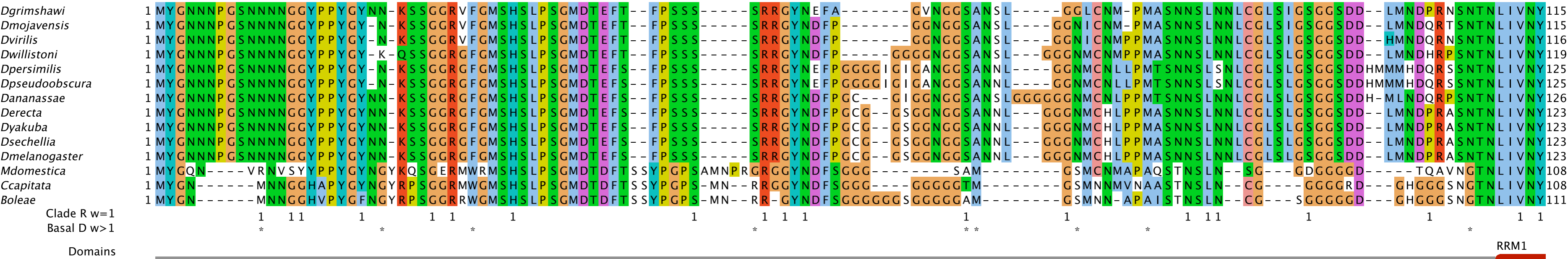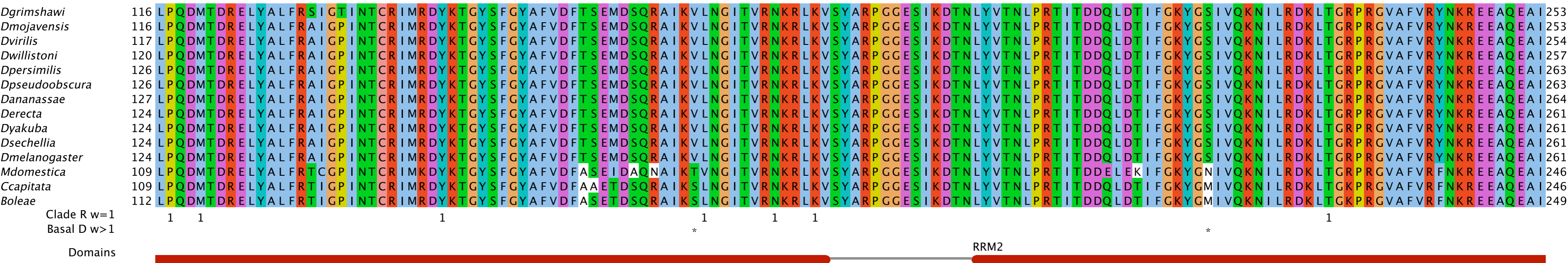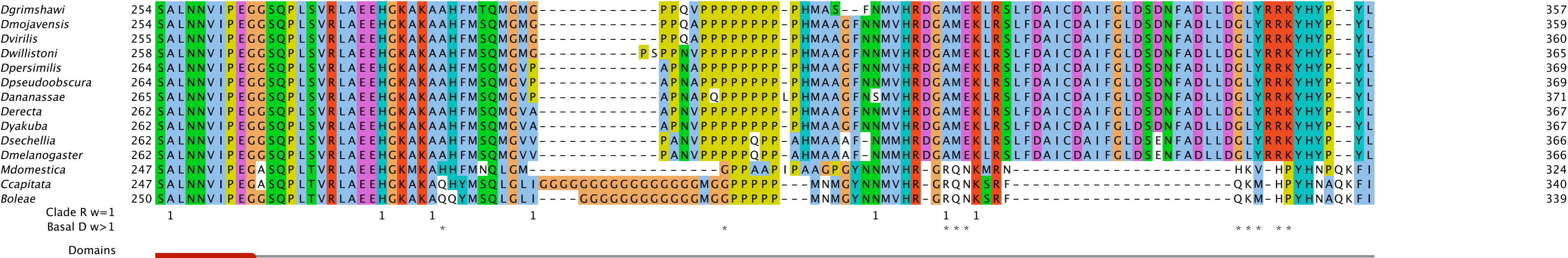

Supplement: Additional file 1 — Figure S1. Alignment of Sex-lethal of Drosophila species, the Tephritidae and M. domestica. Alignment used for analyses of Sex-lethal including sequences from Drosophila species, the Tephritidae and Musca domestica. The alignment is shown translated into amino acids. Sites under relaxed selection in the Tephritidae and Musca are indicated by a "1" in the line "Clade R ω = 1", those under positive selection on the basal brach leading to Drosophila Sex-lethal are indicated by a "*" in the line "Clade Droso ω > 1". These site-specific results are based on Bayes Empirical Bayes analyses mentioned in the main text. The RRM domains of the protein are also shown. [file 1471-2148-12-5-S1.PDF]
