# Supplementary material for: Molecular evolution of Drosophila Sex-lethal and related sex determining genes
Source: BMC Evol Biol. 2012 Jan 14;12:5. doi: 10.1186/1471-2148-12-5 (PMC3292462; doi:10.1186/1471-2148-12-5)
Supplement: Additional file 2 — Table S1. Maximum likelihood models of selection on Sxl in Drosophila, the Tephritidae and M. domestica sequences. [file 1471-2148-12-5-S2.PDF]

Table S1. Maximum likelihood models of selection on Sxl in Drosophila, the Tephritidae and M. domestica sequences.

Branch(es)	Model	N of parameters	Log-likelihood
-	One ratio	1	-4540.06
-	Nearly neutral	2	-4483.80
-	Positive selection	4	-4483.80
Basal-*Drosophila*	Local relaxation	4	-4321.44
	Local selection	5	-4316.86
Basal-Tephritidae	Local relaxation	4	-4352.71
	Local selection	5	-4349.98
Drosophila	Local relaxation	4	-4359.67
	Local selection	5	-4359.67
Remainder	Local relaxation	4	-4379.65
	Local selection	5	-4379.65
