# Supplementary material for: Molecular evolution of Drosophila Sex-lethal and related sex determining genes
Source: BMC Evol Biol. 2012 Jan 14;12:5. doi: 10.1186/1471-2148-12-5 (PMC3292462; doi:10.1186/1471-2148-12-5)
Supplement: Additional file 4 — Table S2. Maximum likelihood ratio models for selection on Drosophila and tephritid Sxl and Drosophila ssx. [file 1471-2148-12-5-S4.PDF]

Table S2. Maximum likelihood ratio models for selection on Drosophila and tephritid Sxl and Drosophila ssx.

Branch(es)	Model	N of parameters	Log-likelihood
-	One ratio	1	-7041.53
-	Nearly neutral	2	-6946.92
-	Positive selection	4	-6946.92
Basal-*ssx*	Local relaxation	4	-6917.04
	Local selection	5	-6913.07
Clade-*ssx*	Local relaxation	4	-6850.07
	Local selection	5	-6850.07
