# Supplementary material for: Molecular evolution of Drosophila Sex-lethal and related sex determining genes
Source: BMC Evol Biol. 2012 Jan 14;12:5. doi: 10.1186/1471-2148-12-5 (PMC3292462; doi:10.1186/1471-2148-12-5)
Supplement: Additional file 6 — Table S3. Maximum likelihood models of selection on transformer in Drosophila and the Tephritidae. [file 1471-2148-12-5-S6.PDF]

Table S3. Maximum likelihood models of selection on transformer in Drosophila and the Tephritidae.

Branch(es)	Model	N of parameters	Log-likelihood
-	One ratio	1	-4136.36
-	Nearly neutral	2	-4129.49
-	Positive selection	4	-4129.49
Basal	Local relaxation	4	-4126.80
	Local selection	5	-4124.17
Drosophila	Local relaxation	4	-4097.05
	Local selection	5	-4097.05
Tephritidae	Local relaxation	4	-4121.60
	Local selection	5	-4121.60
