# Supplementary material for: Molecular evolution of Drosophila Sex-lethal and related sex determining genes
Source: BMC Evol Biol. 2012 Jan 14;12:5. doi: 10.1186/1471-2148-12-5 (PMC3292462; doi:10.1186/1471-2148-12-5)
Supplement: Additional file 8 — Table S4. Maximum likelihood models of selection on doublesex in Drosophila, the Tephritidae and M. domestica. [file 1471-2148-12-5-S8.PDF]

Table S4. Maximum likelihood models of selection on doublesex in Drosophila, the Tephritidae and M. domestica.

Branch(es)	Model	N of parameters	Log-likelihood
-	One ratio	1	-8211.64
-	Nearly neutral	2	-8119.83
-	Positive selection	4	-8119.83
Basal-*Drosophila*	Local relaxation	4	-8110.65
	Local selection	5	-8105.39
Basal-Tephritidae	Local relaxation	4	-8111.28
	Local selection	5	-8107.11
Drosophila	Local relaxation	4	-8101.51
	Local selection	5	-8101.51
Remainder	Local relaxation	4	-8084.74
	Local selection	5	-8084.74
