# Supplementary material for: Molecular evolution of Drosophila Sex-lethal and related sex determining genes
Source: BMC Evol Biol. 2012 Jan 14;12:5. doi: 10.1186/1471-2148-12-5 (PMC3292462; doi:10.1186/1471-2148-12-5)
Supplement: Additional file 9 — Table S5. GI Accession numbers for sequences. [file 1471-2148-12-5-S9.PDF]

Table S5. GI Accession numbers for sequences.

Gene	Numbers
Sxl	2981304, 52075415.
tra	157930032, 157930030, 157930028, 157930026, 157930024, 157930022, 157930020, 157930012, 157930010, 52075411, 22003420.
dsx	2827982, 2827984, 46019686, 46019688, 62999442, 62999444, 95044935, 95044937, 95044939, 95044941, 95044943, 95044945, 56384904, 56384902, 165934579, 165934086, 95044979, 165934086, 95044979, 95044977, 95044975, 95044973, 95044971, 95044969, 95044929, 95044981, 38564770, 38564768.
