# Supplementary material for: Molecular evolution of Drosophila Sex-lethal and related sex determining genes
Source: BMC Evol Biol. 2012 Jan 14;12:5. doi: 10.1186/1471-2148-12-5 (PMC3292462; doi:10.1186/1471-2148-12-5)
Supplement: Additional file 5 — Figure S3. Alignment of transformer of Drosophila species and the Tephritidae only. Alignment used for analyses of transformer including sequences from Drosophila species and the Tephritidae. The alignment is shown translated into amino acids. Sites under relaxed selection in the tephritid clade are indicated by a "1" in the line "Clade Teph ω = 1", those under relaxed selection in the Drosophila clade are indicated by a "1" in the line "Clade Droso ω = 1". These site-specific results are based on Bayes Empirical Bayes analyses mentioned in the main text. The domains that are conserved in the Tephritids but absent in Drosophila and longer that 10 AAs are from position 2 to 99, 215 to 249, 295 to 317 and 370 to 380 of A. serpentina. [file 1471-2148-12-5-S5.PDF]

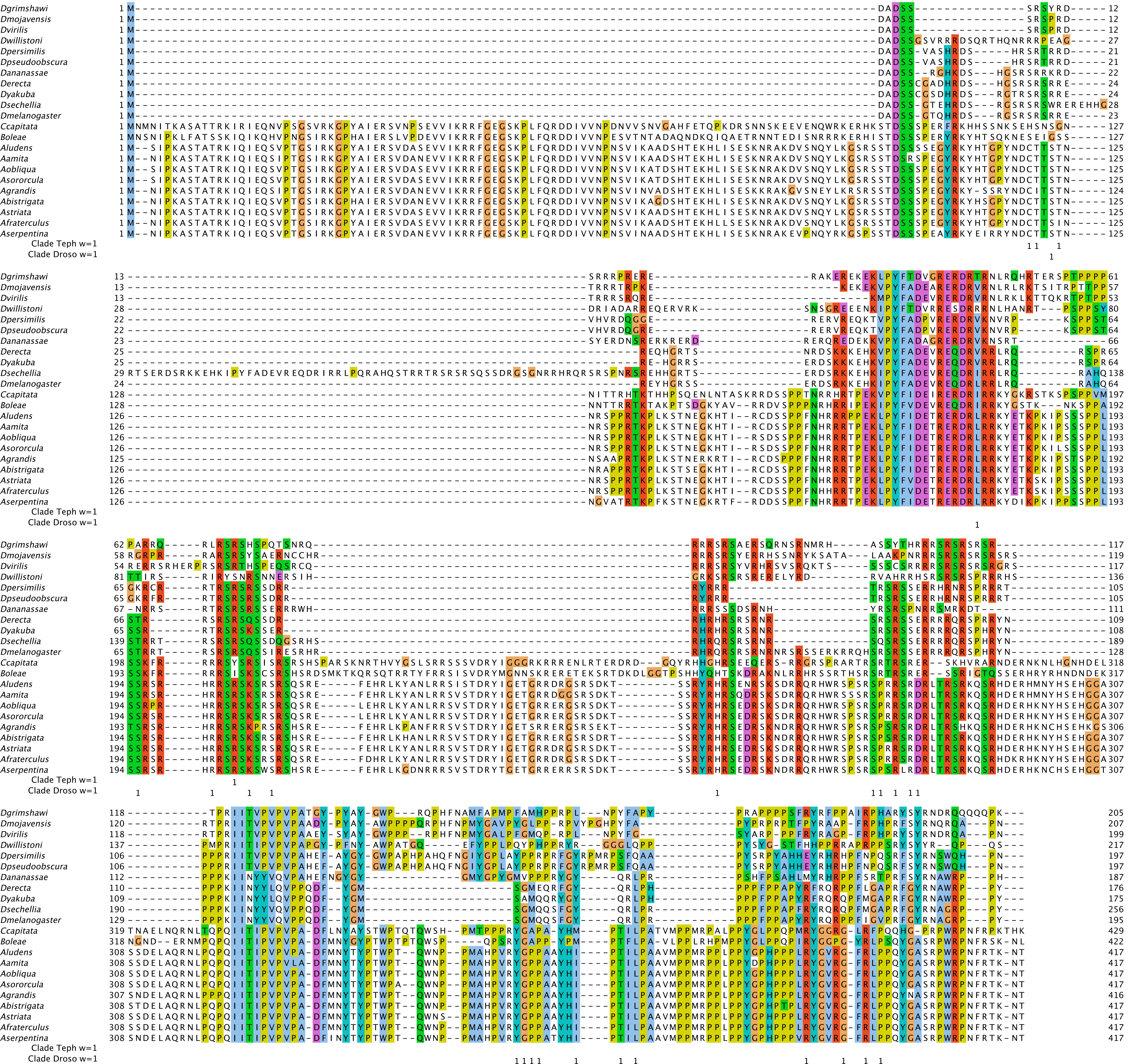
Dgrimshawi
Dmojavensis
Dvirilis
Dwillistoni
Dpersimilis
Dpseudoobscura
Dananassae
Derecta
Dyakuba
Dsechellia
Dmelanogaster
Ccapitata
Boleae
Aludens
Aamita
Aobliqua
Asororcula
Agrandis
Abistrigata
Astriata
Afraterculus
Aserpentina
Clade Teph w=1
Clade Droso w=1
